# Comprehensive analysis of lncRNA-mediated ceRNA regulatory networks and key genes associated with papillary thyroid cancer coexistent with Hashimoto’s thyroiditis

**DOI:** 10.1186/s12902-022-01173-6

**Published:** 2022-10-20

**Authors:** Yuepeng Zhang, Yueli Tian

**Affiliations:** 1 Department of Ultrasound, Zhongnan Hospital of Wuhan University, No. 169, East Lake Road, Wuchang District, Wuhan, Hubei 430071 China; 2 Department of Nuclear Medicine, Zhongnan Hospital of Wuhan University, Wuhan, China

**Keywords:** Papillary thyroid cancer, Hashimoto’s thyroiditis, lncRNA, microRNA, ceRNA

## Abstract

**Objective:**

The incidence of papillary thyroid cancer (PTC) concomitant with Hashimoto’s thyroiditis (HT) is gradually increasing over the past decades. This study aims to identify differentially expressed lncRNAs between tumor tissues of PTC with or without HT and further to confer a better understanding of lncRNA-based competing endogenous RNA (ceRNA) network in PTC with HT.

**Methods:**

GSE138198 containing tissue mRNA data and GSE192560 containing lncRNA data were utilized to perform differentially expression analysis. The ceRNA network was constructed based on miRNA-mRNA interactions merging with lncRNA-microRNA interactions. Functional enrichment analysis and protein–protein interaction (PPI) analysis were performed. The mRNA levels of core genes in the PPI analysis in tumor tissues collected from 112 PTC patients including 35 cases coexistent with HT were determined by quantitative real-time polymerase chain reaction (qRT-PCR).

**Results:**

A total of 57 genes and 40 lncRNAs, with value of |log2 fold change (FC)|≥ 1 and the adjusted *P*-value < 0.05, were deemed as differentially expressed genes and lncRNAs between PTC with and without HT. The pathways most significantly enriched by differentially expressed genes between PTC with and without HT were viral protein interaction with cytokine and cytokine receptor and cytokine-cytokine receptor interaction. CXCL10, CXCL9, CCL5, FCGR3A, and CCR2 owned degree values not less than 10 were deemed as core genes differentially expressed between PTC with and without HT. A total of 76 pairs of lncRNA-miRNA-mRNA ceRNA were obtained. Results of qRT-PCR partially demonstrated the bioinformatics results that the mRNA levels of CXCL10, CXCL9, CCL5, and CCR2 were remarkably elevated in tumor tissues collected from PTC patients coexistent with HT than those without HT (*P* < 0.001).

**Conclusion:**

Our study offers a better understanding of the lncRNA-related ceRNA network involved in PTC with HT, providing novel key genes associated with PTC coexistent with HT.

## Introduction

A significantly increased detection rate of thyroid nodule has been noted over the past three decades, mainly due to the increasing use of diagnostic imaging techniques. Thyroid nodule usually appears non-palpable and asymptomatic, and it is frequently incidentally found in radiological examination of unrelated disease. Although thyroid nodule poses little threat to the health of affected patients, the risk of thyroid malignancy in thyroid nodules remains 10–15% [1]. In 2015, 62,000 populations were estimated to be newly diagnosed with thyroid cancer, ranking the fifth common cancer in women [2]. Thyroid cancer describes a heterogeneous pool of tumors including the predominant papillary thyroid cancer (PTC) subtype owning good survival rates, as well as the poorly differentiated thyroid cancer (PDTC) and anaplastic thyroid cancer (ATC) forms being responsible for most of the disease-related morbidity and mortality [3, 4]. According to the origin of cancer cells, thyroid cancer includes follicular-derived thyroid cancers and neuroendocrine C-cell derived thyroid cancer represented by medullary thyroid cancer, accounting for 1–2% of all thyroid cancers. More than 95% of thyroid cancers are finally diagnosed as differentiated thyroid cancer which is a follicular-derived thyroid cancer [5]. The main cause of thyroid cancer has not been determined, but family history, especially radiation exposure in children and adolescents, including natural radiation, iodine intake, and radiation in treatment and diagnosis are potential risk factors for thyroid cancer [6, 7]. Hashimoto’s thyroiditis, also known as chronic lymphocytic thyroiditis, is a common autoimmune thyroid disease, and its inflammation has been reported to be associated with the occurrence of thyroid cancer. Increasing evidence shows that patients with PTC concomitantly experience Hashimoto’s thyroiditis. Furthermore, a strong correlation has been found between the rising incidence of thyroid cancer and the increase in autoimmune thyroid disease [8–10].

Long noncoding RNAs (lncRNAs) represent a group of transcripts with > 200 nucleotides and have been characterized as crucial regulators of biological processes and tumorigenesis by control of various key cellular genes. Recent studies have established that lncRNAs, such as m6A RNA methylation-related lncRNAs and differentiation-related lncRNAs are deregulated in PTC [11, 12]. An increasing number of microRNAs (miRNAs) with less than 200 nucleotides were found to be abnormally expressed in human thyroid cancer and involved in the development of tumor biology [13, 14]. The hypothesis of competing endogenous RNA (ceRNA) network represents one of attractive paradigms of lncRNA regulation. LncRNAs share microRNA (miRNA) binding sites and modulates posttranscriptional messenger RNA (mRNA) by depletion of miRNA when the ceRNA network is interpreted [15, 16]. A surge of attention has been paid to involvement of lncRNA-based ceRNA network that expedites the development of human diseases including PTC. For example, lncRNA XIST, as ceRNA for miR-34a, regulates cell proliferation and tumor growth of thyroid cancer through the phosphoinositide 3-kinase/protein kinase B (PI3K/AKT) signaling [17]. In a study of papillary thyroid carcinoma, silencing of lncRNA MIAT, acting as ceRNA for miR-150-5p, promoted cell proliferation and migration, and these results were achieved by inhibiting EZH2 (direct downstream target of miR-150-5p) expression [18]. Several lncRNAs such as IFNG-AS1, NR_038461, and T204821 have been shown to play a role in the pathogenesis of autoimmune thyroid disease through modulation of cellular immune response pathways [19]. An increased expression of IFNG-AS1 was observed in patients with Hashimoto's thyroiditis, and there was a positive correlation between the IFNG-AS1 level and the proportion of circulating Th1 cells [20]. However, the value of lncRNAs in the differences in the presence or absence of HT has not yet been investigated. In this study, we attempt to identify differentially expressed lncRNAs between tumor tissues of PTC with or without HT and further to confer a better understanding of lncRNA-based ceRNA action in PTC with HT.

## Methods

### Data sources and processing

We downloaded raw data derived from PTC patients with or without HT from the Gene Expression Omnibus database (GEO, http://www.ncbi.nlm.nih.gov/geo), and these data must be sourced from human PTC tissue samples, profiled by same technology, and supplemented with clear series matrix files and gene symbols. Accordingly, GSE138198 containing tissue mRNA data and GSE192560 containing lncRNA data were eligible for this study and utilized to perform differentially expression analysis. The GSE138198 dataset, processed on the GPL6244 platform and public on Jun 16, 2020, encompasses 6 pieces of PTC without HT and 8 pieces of PTC with HT. The GSE192560 dataset, processed on the GPL16956 platform and public on Mar 09, 2022, encompasses 5 pieces of PTC with or without HT for each. The mRNAs and lncRNAs deemed differentially expressed should fulfill log2-fold change |log2FC|≥ 1 and the adjusted *p*-value < 0.05.

### Functional enrichment analysis

The “clusterProfiler,” “enrichplot,” and “ggplot2” packages in the R environment were employed to retrieve the functional enrichment analysis for genes differentially expressed between PTC background with or without HT, focusing on of Gene Ontology (GO) terms and Kyoto Encyclopedia of Genes and Genomes (KEGG) pathways. The GO analysis applied biologic process (BP), cellular component (CC), and molecular function (MF), to deem gene annotation and gene products attributes. The KEGG analysis offers annotation information with regard to gene signal transduction and disease pathways. The *p*-value less than 0.5 denoted the GO term and pathways significantly enriched by genes, and the top 10 GO terms for each domain and the top 20 KEGG-defined pathways were visualized as bubble plots using the “pathview” package in R software.

### Protein–protein interaction (PPI) analysis

The PPI analysis was carried for genes differentially expressed between PTC background with or without HT using the Search Tool for the Retrieval of the Interacting Genes (STRING) (v11.0) (https://string-db.org/). The PPI network was presented using Cytoscape software (v3.9.0), where the confidence level of node was 0.4, otherwise the sparse genes were removed.

### Construction of the ceRNA network

At first, we mapped the core genes ranking the top 5 in the PPI network into TargetScan (http://www.targetscan.org/), miRDB (http://mirdb.org/), and mirDIP (http://ophid.utoronto.ca/mirDIP/index.jsp) databases to predict miRNA-mRNA interactions. We continued to import the miRNAs in the miRNA-mRNA interactions obtained above into the RNA22 database to identify lncRNA-miRNA interactions. The lncRNAs in the lncRNA-miRNA interactions obtained above were overlapped with lncRNAs differentially expressed between PTC background with or without HT by Venn intersection tool which must follow the principle of ceRNA hypothesis that lncRNAs shared same expression patterns as gene differentially expressed between PTC background with or without HT.

### Patients and tissue samples

This cohort study analyzed retrospective data from 112 consecutive patients undergoing partial or entire thyroidectomies for PTC at Zhongnan Hospital of Wuhan University between January 1, 2021 and December 31, 2021. Fresh-frozen thyroid specimens were obtained from 112 patients. The preoperative diagnosis of TNM stage adopts the 8th edition of American Joint Committee on Cancer/Union for International Cancer Control (AJCC/UICC) tumor-node-metastasis (TNM) staging system for PTC [21]. The inclusion criteria were: i) histopathological examination confirmed with primary PTC with or without coexistence of HT, ii) no earlier history of any treatment for thyroid conditions, iii) no levothyroxine or anti-thyroid drugs were administrated 30 days prior to surgery, and iv) no evidence of immunodeficiency. The exclusion criteria were: i) the presence of other autoimmune diseases in addition to HT, ii) having other pathological types of thyroid malignancies, iii) secondary PTC to other malignancies, or iv) family history of malignant thyroid tumors. The coexistence of HT in PTC was confirmed by two experienced pathologists according to the histological examination of surgical specimens of tumorous thyroid tissues and detections of antithyroglobulin (anti-TGAb) and (TPO-Ab), including the following characteristics no exceptions: i) the histological pathology showed cancer tissues infiltrated and densely packed by a large number of lymphocytes, plasma cells and oxyphilic cells, focal infiltration in the cancer parenchyma, lymphoid follicle formation, and the presence of reactive germinal centers [22]; the infiltrate occurred in a normal region of the thyroid gland not just a peritumoral inflammatory response; ii) the serum concentration of antithyroglobulin (anti-TGAb) ≥ 115 IU/mL; and iii) the serum concentration of antithyroid peroxidase (TPO-Ab) ≥ 34 IU/mL. The concentrations of anti-TGAb and TPO-Ab were determined within a month prior to thyroidectomy using the immune-electrochemiluminescence method. We reviewed the demographic and clinicopathological data of included patients including age, gender, tumor size (the largest diameter for multifocal carcinoma), multifocality, the concentrations of thyroid stimulating hormone (TSH), anti-TGAb and TPO-Ab before surgery, extrathyroidal extension, the presence of central compartment lymph node metastasis (CLNM), and the TNM stage.

### RNA extraction and quantitative real-time polymerase chain reaction (qRT-PCR)

The total RNA of tissue samples was extracted using TRIzol Reagent (Invitrogen, USA) and then utilized as template to generate cDNA by using a PrimeScript RT Reagent Kit (Takara, Dalian, China). The qRT-PCR run on a thermal cycler ABI Prism 7500 using SYBR Green dye based on the manufacturer's recommended protocol (Takara, Dalian, China) to determine expressions of CXCL10, CXCL9, CCL5 and CCR2 relative to GAPDH by adopting comparative cycle threshold values (2^−ΔΔCt^). Primer sequences are listed in Table 1.

**Table 1:** The primer sequences used for qRT-PCR

**Gene**	**Primer sequence (5’-3’)**
CXCL10	Sense: 5’-TCTGACTCCCAAGATTGCCG-3’
	Antisense: 5’-ACTGTGCTAACCTTCTCTGCTG-3’
CXCL9	Sense: 5’-TGGAGGGGGTAACCATCTGA-3’
	Antisense: 5’-CTTTTTCTTTCCCTCAGGAACCC-3’
CCL5	Sense: 5’-ACACTTGACATTGTGCTGGAC-3’
	Antisense: 5’-AGTGGCAACTGATGCTTCCC-3’
CCR2	Sense: 5’-AAGCTGGTGAGTTGTAGGCA-3’
	Antisense: 5’-TAACGTCCTTGGGTGCTCAG-3’

### Statistical analysis

A manner of mean and standard deviation (sd) is used to present continuous variables. Independent t-tests were used for continuous variables. Rate (%) or composition ratio is used to present categorical variables. Statistical tests including unpaired t test and chi-square test were conducted, graphs and figures were produced in the GraphPad Prism 8.0 software (GraphPad Software, La Jolla, CA, USA). *P* < 0.05 denotes a significant difference.

## Results

### Identification of genes and lncRNAs differentially expressed between PTC with and without HT

**Fig. 1:**
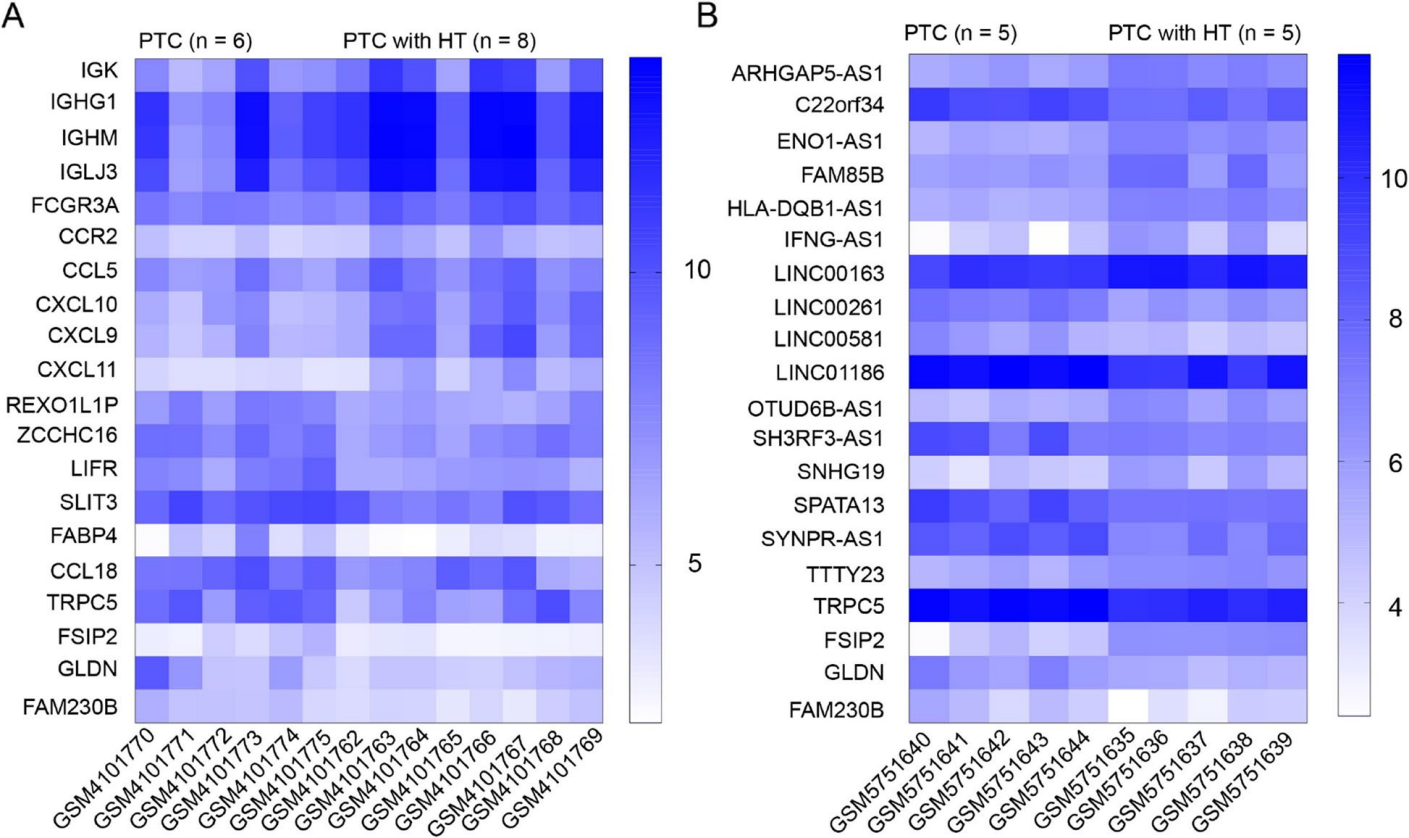
The heatmaps showing expression diversity of genes (**A**) and lncRNAs (**B**) differentially expressed between PTC with and without HT

The mRNA and lncRNA data deposited in the GSE138198 and GSE192560 datasets were differentially analyzed, and those with value of |log2FC|≥ 1 and the adjusted *p*-value < 0.05 were deemed as differentially expressed genes and lncRNAs between PTC with and without HT. Accordingly, we acquired 57 genes differentially expressed between PTC with and without HT, including 43 upregulated genes and 14 downregulated ones, and 40 lncRNAs differentially expressed between PTC with and without HT, including 13 upregulated lncRNAs and 27 downregulated ones (Fig. 1A, B).

### Enrichment analysis of genes differentially expressed between PTC with and without HT

**Fig. 2:**
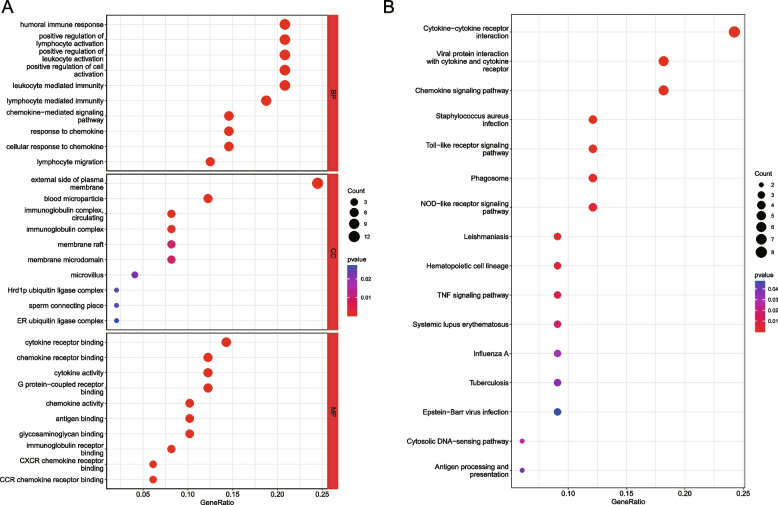
The top 10 most enriched GO terms at the BP, CC, and MF domains (**A**) and the 16 enriched KEGG pathways [23] (**B**) were visualized as bubble plots. Larger circles reflect more gene enriched, and bluer indicates smaller p values

We then conducted GO annotation and KEGG pathway analyses to evaluate the main functional pathways associated with 57 genes differentially expressed between PTC with and without HT. After GO analysis, it was found there were 489 GO terms significantly enriched by these 57 genes (*P* < 0.05), including 426 terms belonging to the BP domain, 18 terms belonging to the CC domain, and 45 terms belonging to the MF domain. The most enriched GO terms at the BP domain were “chemokine-mediated signaling pathway”, followed by “response to chemokine”, and then “cellular response to chemokine”, while the most enriched GO terms in the CC and MF domains were “external side of plasma membrane” and “chemokine receptor binding”, respectively. The top 10 GO terms for each domain are presented in Fig. 2A. After KEGG pathway analysis, it was found there were 16 KEGG pathways significantly enriched by these 57 genes (*P* < 0.05) (Fig. 2B). The most enriched KEGG pathways were “viral protein interaction with cytokine and cytokine receptor” and “cytokine-cytokine receptor interaction”.

### Identification of core genes differentially expressed between PTC with and without HT

**Fig. 3:**
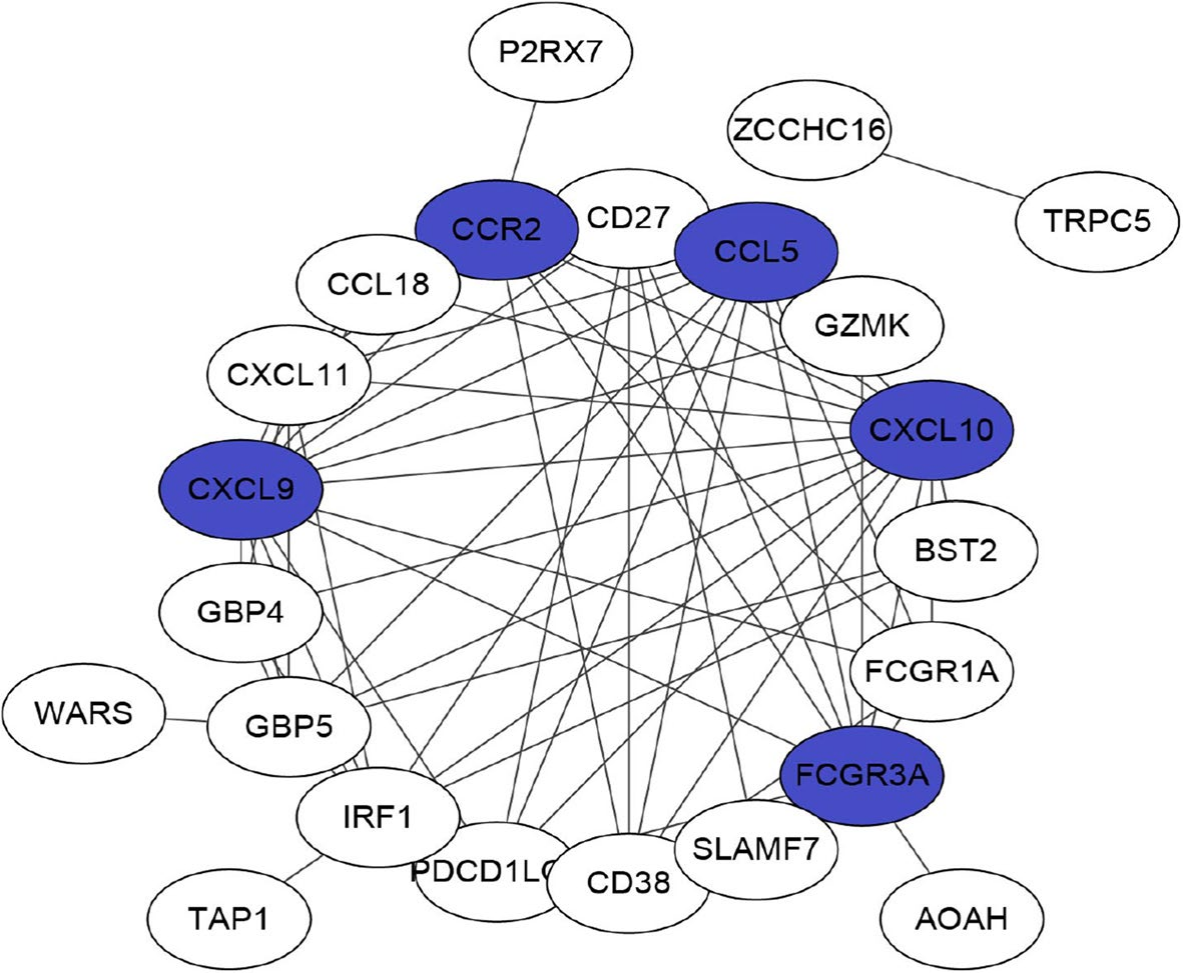
Construction of PPI network. The PPI network encompasses 23 nodes with 70 interactions, among which CXCL10, CXCL9, CCL5, FCGR3A, and CCR2 owned degree values not less than 10 were deemed as core genes differentially expressed between PTC with and without HT

We subsequently conduct PPI analysis by importing genes differentially expressed between PTC with and without HT into the STRING database. Figure 3 presents the PPI network in which 23 nodes with 70 interactions were characterized. Chemokines CXC chemokine ligand (CXCL) 10, CXCL9, CC chemokine ligand 5 gene (CCL5), Fcgamma receptor 3A (FCGR3A), and CC chemokine receptor 2 (CCR2) owned degree values not less than 10 were deemed as core genes differentially expressed between PTC with and without HT, all which were upregulated in tumor tissue samples of PTC with HT compared with those without HT.

### Final construction of the ceRNA network associated with PTC with HT

**Fig. 4:**
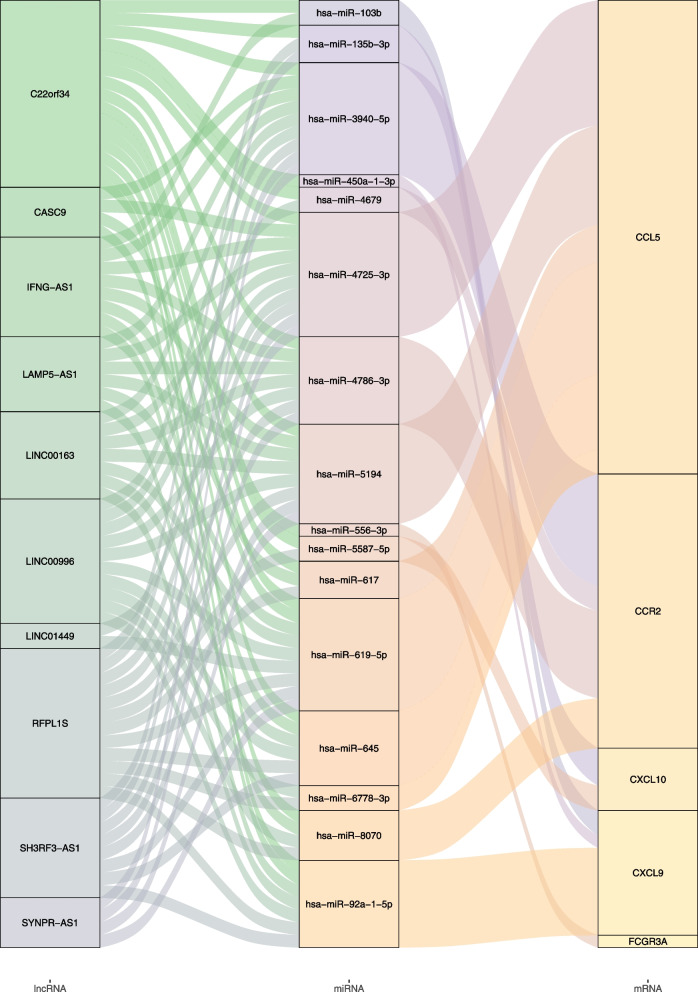
Final construction of ceRNA network in RIF

We searched the TargetScan, miRDB, and mirDIP databases for putative miRNAs based on CXCL10, CXCL9, CCL5, FCGR3A, and CCR2. There were two miRNAs targeting CXCL10, three miRNAs targeting CXCL9, six miRNAs targeting CCL5, a miRNA targeting FCGR3A, and four miRNAs targeting CCR2. After removing the overlapping these miRNAs, miR-617, miR-619-5p, miR-645, miR-4725-3p, miR-5194, miR-6778-3p, miR-8070, miR-4786-3p, miR-4679, and miR-3940-5p were imported into the RNA22 database to obtain lncRNAs interaction with these miRNAs. These putative lncRNAs were overlapped with 40 lncRNAs differentially expressed between PTC with and without HT. On the basis of the principle of ceRNA hypothesis, the overlapped lncRNAs shared same expression patterns as CXCL10, CXCL9, CCL5, FCGR3A, and CCR2 were selected to construct the ceRNA network associated with PTC coexistent with HT. A total of 76 pairs of lncRNA-miRNA-mRNA ceRNA were obtained, including 15 pairs based on C22orf34, 12 pairs based on RFPL1S, and 10 pairs based on LINC00996 (Fig. 4).

### Clinical validation

**Fig. 5:**
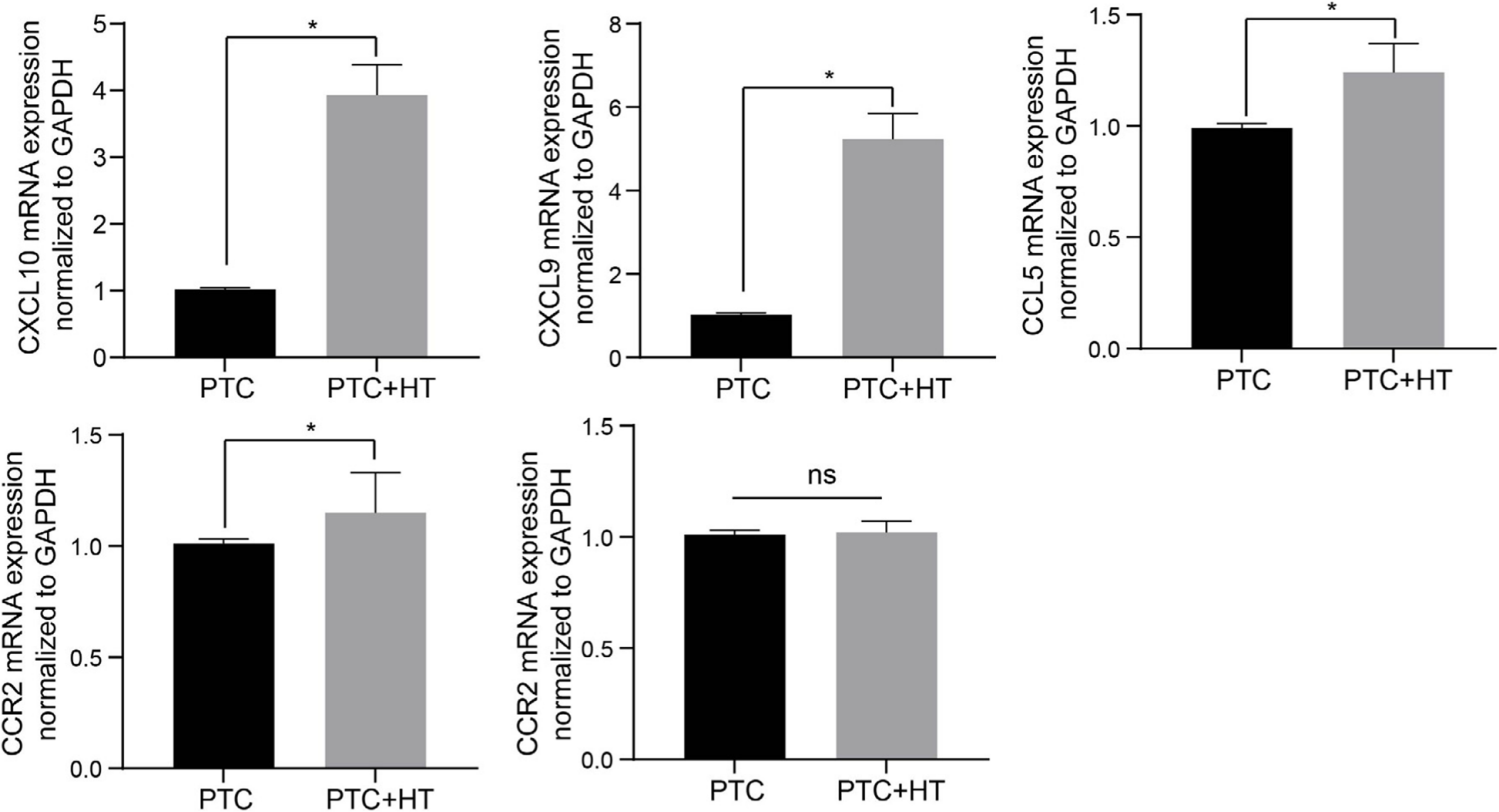
The mRNA levels of CXCL10, CXCL9, CCL5, CCR2, and FCGR3A in tumor tissues collected from PTC patients coexistent with HT than those without HT were determined by qRT-PCR. *P* < 0.001. ns indicates no significance

We collected tumor tissue specimens from 112 consecutive patients undergoing partial or entire thyroidectomies for PTC. According to the results of postoperative pathological examinations, detection of anti-TGAb and TPO-Ab concentrations, there were 35 PTC patients coexistent with HT. As shown by Table 2, PTC patients with or without HT did not exhibit statistically considerable difference in light of age, gender, tumor size, multifocality, extrathyroidal extension, the presence of CLNM, and the TNM stage (*P* > 0.05). PTC patients with HT had higher concentrations of TSH, anti-TGAb, and TPO-Ab than those without HT before surgery (*P* < 0.05). Results of qRT-PCR partially demonstrated the bioinformatics results that the mRNA levels of CXCL10, CXCL9, CCL5, and CCR2 were remarkably elevated in tumor tissues collected from PTC patients coexistent with HT than those without HT (Fig. 5, *P* < 0.001), while the mRNA level of FCGR3A did not differ (*P* > 0.05).

**Table 2:** The demographic and clinical characteristics of patients with HT in PTC background

**Variables**	**PTC (*n* = 112)**		***P***
	**HT absence (*n* = 77)**	**HT presence (*n* = 35)**	
Age (year, mean ± sd)	43.64 ± 11.49	42.06 ± 13.76	0.528
Gender [n(%)]			0.751
Male	9 (11.69%)	3 (8.60%)	
Female	68 (88.31%)	32 (91.40%)	
Tumor size (cm, mean ± sd)	1.28 ± 0.72	1.09 ± 0.50	0.161
Multifocality [n(%)]			0.674
Positive	27 (35.06%)	14 (40.00%)	
Negative	50 (64.94%)	21 (60.00%)	
TSH (mIU/L, mean ± sd)	2.43 ± 1.45	3.11 ± 1.75	0.034
Anti-TGAb	24.27 ± 13.55	173.17 ± 137.53	< 0.001
TPO-Ab	38.26 ± 15.57	525.44 ± 533.05	< 0.001
Extrathyroidal extension [n(%)]			0.172
Positive	23 (29.87%)	6 (17.14%)	
Negative	54 (70.13%)	29 (82.86%)	
CLNM [n(%)]			0.834
Positive	30 (38.96%)	15 (42.86%)	
Negative	47 (61.04%)	20 (57.14%)	
TNM stage 8th [n(%)]			0.533
I, II	76 (98.68%)	34 (97.14%)	
III, IV	1 (1.32%)	1 (2.86%)	

*PTC* Papillary thyroid carcinoma, *HT* Hashimoto’s thyroiditis, *TSH* Thyroid stimulating hormone, *anti-TGAb* Antithyroglobulin antibody, *TPO-Ab* Thyroid peroxidase antibody, *CLNM* Central compartment lymph node metastasis, *TNM* Tumor node metastasis

## Discussion

The prevalence of PTC coexistent with HT has increased continuously and the relationship between two of them has received much attention [24]. Previous evidence has shown the incidence of PTC in HT patients is 1.3–2 times higher than those with thyroid benign diseases in the absence of HT [25]. However, another evidence has emerged that PTC patients coexistent with HT are more likely to have favorable prognosis as less invasive disease at presentation and a lower recurrence rate were observed [26]. This observation is rational concerning the mechanism that lymphocyte infiltration resulting from HT may cause antitumor immunity [27, 28]. However, relevant evidence is lacking in most multivariate analyses, which makes a definite association between PTC and HT is still a matter of debate [29]. Molecular, hormonal and histopathalogical basis of this association requires better interpretation. The aim of this study was to identify differentially expressed lncRNAs between tumor tissues of PTC with or without HT and further to confer a better understanding of lncRNA-based ceRNA action in PTC with HT. An exploration of key genes between tumor tissues of PTC with or without HT may provide a more precise strategy for the diagnosis and treatment of PTC of different types and characteristics, particularly PTC with HT.

Our results obtained by bioinformatics methods characterized a total of 76 pairs of lncRNA-miRNA-mRNA ceRNA associated with PTC coexistent with HT, including 15 pairs based on C22orf34, 12 pairs based on RFPL1S, and 10 pairs based on LINC00996. On the basis of the principle of ceRNA hypothesis, C22orf34, RFPL1S, and LINC00996 shared same expression patterns as CXCL10, CXCL9, CCL5, FCGR3A, and CCR2, all which were upregulated in tumor tissues of PTC with HT compared with those without HT. A possible association between the single nucleotide variant rs35198919 in intron 1 of the C22orf34 and interstitial lung disease that may be associated with autoimmune disorders [30, 31]. C22orf34 was reported to be a lncRNA signature to predict prognosis and immune response in diffuse large B-cell lymphoma [32]. RFPL1S was identified as 6-kb noncoding antisense mRNAs of RFPL1S antisense gene in 199 by Seroussi et al. [33], whose role in human disease requires further better investigation. LINC00996 was associated with immune cell infiltration status and immunotherapy responses in head and neck squamous cell carcinoma [34].

Multiple cytokines exert a dangerous influence on the body by amplifying inflammatory reactions, causing a series of autoimmune diseases including HT [35, 36]. In addition to autoimmune thyroid disease, cytokines are concerned about their contributions to tumor growth and metastasis in PTC [37]. lncRNAs are believed to function as important regulators of cytokine regulation by the ceRNA phenomenon in PTC complicated with HT [38]. As evidenced by our study, CXCL10, CXCL9, CCL5, and CCR2 as key cytokines were associated with the presence of HT in PTC, all which involved in the C22orf34-, RFPL1S-, and LINC00996-based ceRNA network. Results of qRT-PCR partially demonstrated the bioinformatics results that the mRNA levels of CXCL10, CXCL9, CCL5, and CCR2 were remarkably elevated in tumor tissues collected from PTC patients coexistent with HT than those without HT. The initiation and perpetuation of chronic autoimmune inflammation depends on the recruitment, trafficking, and in situ maintenance of specific subsets of activated lymphocytes [39]. IFN-γ stimulation leading to the release of CXCR3-binding chemokines from thyrocytes in turn recruits Th1 lymphocytes expressing CXCR3 and producing IFN-γ, which suggests that the interferon-γ inducible chemokines (CXCL9, CXCL10, and CXCL11) and their receptor CXCR3 play an important role in the initiation of autoimmune thyroid diseases [40]. CXCL10 and CXCL9 as two interferon(IFN)γ-dependent chemokines of C-X-C chemokine receptor (CXCR)3 are implicated in the immune-pathogenesis of autoimmune thyroiditis [41]. A previous study found that serum CXCL10 levels have been shown to be elevated in autoimmune thyroid diseases [42]. The TSH-lowering effect of selenium supplementation is unlikely to be related to changes in humoral markers of autoimmunity and/or circulating CXCL9 [43]. In vitro experiments showed that the expressions of CCL5 and migration of peripheral blood mononuclear cells were markedly increased, while the level of PPARγ was significantly decreased after the lentivirus-mediated knockdown of Cav-1 in Nthy-ori 3–1 cells [44].

Extensive attention has been paid to PTC and coexistent HT to elucidate their association. In this study, we collected 112 PTC patients including 35 cases with HT. PTC patients with or without HT did not exhibit statistically considerable difference in light of age, gender, tumor size, multifocality, extrathyroidal extension, the presence of CLNM, and the TNM stage. Given relatively small sample size, our data were insufficient to support the concept of HT as a protective or risk factor for PTC. However, we found that PTC patients with HT had higher concentrations of TSH, anti-TGAb, and TPO-Ab than those without HT before surgery. A gradual increase of TSH is common in chronic lymphocytic thyroid autoimmunity. TSH is not only an endogenous stimulator of thyroid hormone production but also a growth factor for thyrocytes, serum TSH elevations may be associated with increased risk of PTC [45]. Accordingly, HT brings a chronic inflammatory condition that awakens an immune response leading to a continuous damage of surrounding stromal cells, leading to an inappropriate cell proliferation and thus increasing the risk of neoplastic transformation [46]. On the opposite, it was reported that PTC patients with HT may have favorable clinicopathologic characteristics compared to PTCs without HT [47]. This inconsistency may result from low mortality rate associated with PTC and the protective effect of HT not being independent of tumor characteristics.

There are two limitations needed to inform when interpreting our results. On the one hand, the sample size of clinical human tissues for RT-qPCR validation of core genes was relatively small, which may weaken the reliability of clinical data. In the future, we will collect more samples to perform RNA-sequencing or arraying for better clinical validation. On the other hand, given the preliminary nature of our study, further functional studies elaborating the specific signal mechanism of CXCL10, CXCL9, CCL5, and CCR2 in cellular and animal models of PTC when coexistent with HT were required to elucidate the pathogenesis of PTC coexistent with HT focusing on lncRNA-based ceRNA action.

## Conclusion

In conclusion, our results indicate that the lncRNA-based ceRNA network might provide new insights from the perspective of RNA for obtaining a further understanding of the clinical features related to PTC with HT. The CXCL10, CXCL9, CCL5, and CCR2 were associated with the presence of HT in PTC. These chemokines may facilitate immunotherapy in PTC when coexistent with HT.

## Data Availability

GSE138198 and GSE192560 datasets were downloaded from the Gene Expression Omnibus database (GEO, http://www.ncbi.nlm.nih.gov/geo) that is a public database. Other data used to support the findings of this study are included within the article.
